# Perceptions of the neighbourhood environment and self rated health: a multilevel analysis of the Caerphilly Health and Social Needs Study

**DOI:** 10.1186/1471-2458-7-285

**Published:** 2007-10-09

**Authors:** Wouter Poortinga, Frank D Dunstan, David L Fone

**Affiliations:** 1 Welsh School of Architecture, Cardiff University, Bute Building, King Edward VII Avenue, Cardiff, Wales, CF10 3NB, UK; 2 Department of Primary Care and Public Health, Centre for Health Sciences Research, Cardiff University, Neuadd Meirionnydd, Heath Park, Cardiff, Wales, CF14 4YS, UK

## Abstract

**Background:**

In this study we examined whether (1) the neighbourhood aspects of access to amenities, neighbourhood quality, neighbourhood disorder, and neighbourhood social cohesion are associated with people's self rated health, (2) these health effects reflect differences in socio-demographic composition and/or neighbourhood deprivation, and (3) the associations with the different aspects of the neighbourhood environment vary between men and women.

**Methods:**

Data from the cross-sectional Caerphilly Health and Social Needs Survey were analysed using multilevel modelling, with individuals nested within enumeration districts. In this study we used the responses of people under 75 years of age (n = 10,892). The response rate of this subgroup was 62.3%. All individual responses were geo-referenced to the 325 census enumeration districts of Caerphilly county borough.

**Results:**

The neighbourhood attributes of poor access to amenities, poor neighbourhood quality, neighbourhood disorder, lack of social cohesion, and neighbourhood deprivation were associated with the reporting of poor health. These effects were attenuated when controlling for individual and collective socio-economic status. Lack of social cohesion significantly increased the odds of women reporting poor health, but did not increase the odds of men reporting poor health. In contrast, unemployment significantly affected men's health, but not women's health.

**Conclusion:**

This study shows that different aspects of the neighbourhood environment are associated with people's self rated health, which may partly reflect the health impacts of neighbourhood socio-economic status. The findings further suggest that the social environment is more important for women's health, but that individual socio-economic status is more important for men's health.

## Background

There is growing awareness among health researchers and policy makers that the local environment is important for people's health. This has resulted in a large number of research initiatives examining the health effects associated with different social and physical aspects of the neighbourhood environment. According to Yen and Syme, this research can be divided into studies investigating the roles of (1) the quality of the local environment, (2) the social organisation of the local community, often reflected in social capital and/or social cohesion, and (3) the socio-economic status of the neighbourhood [1]. All three aspects of the neighbourhood environment appear to be associated with community health. Studies focussing on the *quality of the local environment *have shown that perceived neighbourhood problems and crime, the absence of green space, and poor access to amenities are linked with the reporting of poor health [2-10]. The importance of *social capital and social cohesion *for people's physical and psychological well-being is now well known in the social epidemiological literature [11-13]. Although there is still considerable disagreement about the definition, measurement, and workings of social capital and cohesion, various studies have shown that these complex social constructs are associated with a wide range of health outcomes, such as life-expectancy, mortality rates, and self rated health [14-19]. The group of studies investigating the health effects of community *socio-economic status *consistently report significant associations with a large number of health outcomes, generally showing that people living in deprived neighbourhoods have poorer health [1,20].

A limitation of many studies investigating the health effects of social and physical aspects of the neighbourhood environment is that they fail to take into account differences in neighbourhood socio-economic status. The results of these studies are ambiguous considering that disadvantaged neighbourhoods may suffer from poorer physical and social infrastructures, [5] and that there is still a lack of clarity about the specific pathways that link area of residence to health [21]. It may be that, as suggested by Stafford and Marmot [22], the association between neighbourhood deprivation and health is mediated by perceived neighbourhood and housing problems, or that the associations between specific neighbourhood attributes and health are spurious, and may actually reflect the impact of neighbourhood socio-economic status.

There is some evidence that the place where a person lives may not affect all people in the same way [22-25]. Recent research suggests that the neighbourhood environment is more important for women than for men [26-28]. The social environment appears to be particularly important for women's health status. In contrast, men's health status seems to be more affected by the physical environment and by neighbourhood socio-economic status [28-31].

In this study we examine the importance of different social and physical aspects of the neighbourhood environment for people's self rated health. We specifically focus on the neighbourhood aspects of access to amenities, neighbourhood quality, neighbourhood disorder, and social cohesion; and investigate whether the possible health effects reflect differences in socio-demographic composition and/or neighbourhood deprivation. We additionally examine whether the associations with different aspects of the neighbourhood vary between men and women.

## Methods

### Population

In this study we used population survey data from the Caerphilly Health and Social Needs Study (CHSNS) that were collected in 2001. Caerphilly county borough is situated in south-east Wales, United Kingdom, with an adult population of around 130,000, and is one of five unitary authority local government areas within the former Gwent Health Authority area. The survey sample was drawn from the general practitioner age-sex register held by the former Gwent Health Authority and stratified by census ward. In total, 22,290 questionnaires were distributed. Of these, 12,408 were returned, which equates to a 62.7% response rate after the removal of incorrect addresses. In this study we only use the responses of people under 75 years of age (n = 10,892), as the individual responses for older respondents are less reliable due to cognitive decline. The response rate for this subgroup was 62.3%. All individual responses were geo-referenced to the 325 census enumeration districts (ED) of Caerphilly borough council. EDs were considered the most appropriate geographical unit for this study, as these are the smallest and most homogeneous geographical unit available at the time the work was being carried out. The survey was granted ethical approval by Gwent Local Research Ethics Committee and participant gave implied consent after reading information about the project. A more detailed description of the process of the survey has been given elsewhere [32,33].

### Measures

Self rated health was used as the outcome variable (see Table 1). Respondents were asked to rate their health on a five-point scale. The scale was dichotomised with 1 representing fair and poor health and 0 representing good, very good or excellent health.

**Table 1:** Characteristics of respondents to the Caerphilly Health and Social Needs Study (CHSNS)

		***Men (n = 4,877)***	***Women (n = 6,015)***
**Health Outcome**			
*Self Rated Health*	Excellent/(Very) Good	3079 (63.1%)	4029 (67.0%)
	Fair/Poor	1770 (36.3%)	1939 (32.2%)
	Missing values	29 (0.6%)	47 (0.8%)
			
**Socio-demographics**			
*Age*		Mean= 48.3; SD = 15.2	Mean= 46.4; SD = 15.2
*Social class*	Class I and II	1143 (23.4%)	1276 (21.2%)
	Class III NM	420 (8.6%)	1701 (28.3%)
	Class III M	1705 (35.0%)	501(8.3%)
	Class IV and V	1109 (22.7%)	1588 (26.4%)
	Other	177 (3.6%)	495 (8.2%)
	Missing values	323 (6.6%)	454 (7.5%)
*Employment Status*	Employed	2644 (54.2%)	2903 (48.3%)
	Unemployed	176 (3.6%)	113 (1.9%)
	Retired	999 (20.5%)	1190 (19.8%)
	Disability	669 (13.7%)	647 (10.8%)
	Other	160 (3.3%)	841 (14.0%)
	Missing values	229 (4.7%)	321 (5.3%)
*Household Tenure*	Home owner	3980 (81.6%)	4723 (78.5%)
	Non home owner	822 (16.9%)	1188 (19.8%)
	Missing values	75 (1.5%)	104 (1.7%)
			
**Neighbourhood perceptions **^**(1)**^			
*Access to amenities *(scale 1–5)		Mean = 3.71; SD = 0.79	Mean = 3.75; SD = 0.82
*Neighbourhood quality *(scale 1–3)		Mean = 1.90; SD = 0.47	Mean = 1.89; SD = 0.47
*Neighbourhood disorder *(scale 1–3)		Mean = 1.56; SD = 0.42	Mean = 1.57; SD = 0.42
*Social cohesion *(scale 1–5)		Mean = 3.63; SD = 0.67	Mean = 3.67; SD = 0.69

(1) Higher scores represent a more negative evaluation of the neighbourhood.

The socio-demographics of gender, age, social class, employment status, and household tenure were included as independent variables in the analyses. Age was considered a continuous variable, and centred at the mean of 47.3 years. Social class was measured using the Registrar General's occupation-based classification: I and II (professionals and intermediates), III NM (skilled non-manual), III M (skilled manual), IV and V (partly and unskilled manual), and 'other' (e.g., armed forces). The employment status variable had five categories: employed, unemployed, retired, disabled, and other (i.e., student and looking after home or children). Household tenure compared homeownership (with or without mortgage) with non-homeownership (renting from housing association/trust or private landlord and living rent free).

The neighbourhood perception variables that were considered in this study were: (1) *access to amenities*, (2) *neighbourhood problems*, and (3) *social cohesion*.

*Access to amenities *was measured by asking respondents how well placed their home is for ten amenities, i.e., getting to work, job opportunities, food stores with fresh fruit and vegetables, your doctor's surgery, the nearest hospital with a casualty department, schools, libraries, public transport, general shopping, and leisure facilities. People could respond using a five-point scale with the following categories: 1: "very well placed", 2: "fairly well placed", 3: "average", 4: "not very well placed", and 5: "not at all well placed". Because the first two items had large numbers of missing values they were omitted from further analyses. We summed the remaining eight items, which formed an internally consistent scale (Cronbach's α = 0.86).

*Neighbourhood problems *were measured by asking people to indicate how much of a problem 14 issues are "in this area" [34]. People could respond using a three-point scale: 1: "not a problem", 2: "some problem", and 3: "serious problem". A principal components analysis with Varimax rotation produced two clearly interpretable components. The first component included 'social nuisances', such as: litter and rubbish, uneven and dangerous pavements, nuisance from dogs, and noise. The second component reflected more serious crime in the neighbourhood, such as: vandalism, assaults and muggings, burglaries, and discarded needles and syringes. The components were labelled as *neighbourhood quality *and *neighbourhood disorder*, respectively. The items that loaded highly on the two respective components were summed to form internally consistent scales (Cronbach's α = 0.78 and 0.77, respectively).

*Social cohesion *was measured by asking respondents to what extent they agree with eight statements derived from an adapted version of Buckner's Neighbourhood Cohesion scale [35]: "I visit my friends in their homes", "The friendships and associations I have with other people in my neighbourhood mean a lot to me", "If I need advice about something I could go to someone in my neighbourhood", "I believe my neighbours would help in an emergency", "I borrow things and exchange favours with my neighbours", "I would be willing to work together with others on something to improve my neighbourhood", "I rarely have a neighbour over to my house to visit", and "I regularly stop and talk with people in my neighbourhood". A five-point response scale was used, with the following categories 1: "strongly agree", 2: "agree", 3: "neither agree nor disagree", 4: "disagree", and 5: "strongly disagree". Responses to the eight statements were summed, forming an internally consistent scale (Cronbach's α = 0.81) [19,36].

The individual neighbourhood perception scales were transformed into four contextual variables at the neighbourhood (ED) level. As the neighbourhood perception scales may have a multilevel structure of their own [37], we conducted separate two-level random-coefficient regression analyses for each of the four neighbourhood perception scales. The four individual neighbourhood perception scales were fitted in turn as dependent variables, and were assumed to have a normal distribution. The models were 'null' with no independent variables. The analyses showed that 23.2%, 15.0%, 16.2%, and 1.8% of the variation could be found at the neighbourhood level for access to amenities, neighbourhood quality, neighbourhood disorder and social cohesion respectively. This suggests that there is more inter-rater agreement about the first three neighbourhood characteristics than there is about neighbourhood social cohesion. We included the standardised values (Z-scores) of the level 2 residuals in the subsequent analyses as neighbourhood-level variables. The level 2 residuals of the four neighbourhood perception variables reflect the neighbourhood-specific contributions to the overall scores [38]. The shrunken residuals derived from multilevel modelling provide more precise estimates of neighbourhood characteristics than aggregated individual variables, as it is based on the part of the variation that may be attributed to the true variation between neighbourhoods [39].

In this study we use the proportion of households with an annual gross income of under £10,000 as an indicator of *neighbourhood deprivation*. The cut-off point was chosen to represent the line of poverty [40]. In the absence of routinely available income data at ED level, we used validated gross household income estimates from a commercial dataset (CACI Ltd's Paycheck Household Income Model) [41,42]. The Paycheck model of neighbourhood income was found to be highly correlated with the widely-used Townsend index of social and material deprivation across the 325 census EDs of Caerphilly borough (r = 0.80). The percentage of households in the 325 Caerphilly EDs with an income under £10,000 ranged from 3.8 to 73.7 (Mean = 31.3; SD = 13.0). The high percentage of low income households in some EDs reflect the high levels of socio-economic deprivation in the borough; two census wards situated in the Upper Rhymney Valley in the north of the borough are among the 5% highest ranking wards in England and Wales on both the Breadline Britain and the Work Poverty Indices [43]. The neighbourhood deprivation variable was standardised by calculating the Z-scores.

### Analysis

Multilevel modelling was utilised to analyse the CHSNS dataset, with individuals as level 1 and EDs as level 2 units. A series of two-level logistic regression models were constructed with self rated health as the dependent variable. The parameters were estimated using Markov Chain Monte Carlo (MCMC) procedures in MLwiN software [44]. First, we estimated the coefficients of the different individual socio-demographic variables in a single model (Model 1). Second, we estimated the coefficients of the different neighbourhood variables in three series of models (Models 2a-c). In Model 2a we estimated the unadjusted associations of the five neighbourhood variables with self-rated health in separate models. In Model 2b we estimated the adjusted coefficients of the neighbourhood variables in five separate models, controlling for the individual socio-demographic variables. In Model 2c we again estimated the adjusted coefficients of the neighbourhood variables in four separate models, but now additionally controlling for neighbourhood deprivation.

We further examined whether there are gender differences in the associations between the neighbourhood environment and self rated health, by estimating the socio-demographic-adjusted coefficients for men and women separately. Possible interactions were later validated by remodelling the complete dataset with the relevant gender interaction terms. That is, the gender interactions were tested in the combined dataset, and adjusted for differences in age, social class, employment status, and home ownership.

In this study several respondents had missing values on one or more of the social class, employment status and housing tenure variables (see Table 1). We dealt with these missing data through multiple imputation [45]. In multiple imputation, missing values are replaced with plausible values a number of times, to reflect the uncertainty about the true values of the missing data. In this study we imputed missing values based on the distribution of the values for the three variables in twelve age-sex strata (using the age groups of 18–24, 25–34, 35–44, 45–54, 55–64, and 65–74 for men and women separately). We repeated this imputation procedure 10 times. We analysed the 10 resulting datasets separately, and subsequently combined the results into one estimate. Standard errors were based on the standard errors from each set of imputed data combined with inter-imputation variation.

## Results

Table 1 presents the characteristics of the CHSNS dataset for men and women separately. It shows that men were slightly more likely to report poor health than women. A gender difference was found for social class: men were more often classified as III M (skilled manual), and women more often as III NM (skilled non-manual). There were also some small differences with regard to employment status: whereas men were more likely to be employed, women were more likely to be a student or to look after the home and/or children. Men and women appeared to have similar perceptions of the neighbourhood environment.

Table 2 shows the bivariate correlations between the different neighbourhood variables and self rated health. Neighbourhood quality, neighbourhood disorder and deprivation had the strongest associations with poor health at the neighbourhood level. Neighbourhood deprivation, neighbourhood quality, and neighbourhood disorder were strongly interrelated, suggesting that they may measure the same thing or are part of the same underlying social processes. The associations between access to amenities and social cohesion on the one hand and poor health on the other were weaker but still significant. Access to amenities had the lowest correlations with the other neighbourhood variables. Its associations with neighbourhood disorder and social cohesion were non-significant.

**Table 2:** Ecological correlations between the different neighbourhood variables and self rated health (n = 325 EDs) ^(1)^

	**Neighbourhood quality**	**Neighbourhood disorder**	**Social cohesion**	**Deprivation**	**Poor health**^**(2)**^
Access to amenities	0.19	0.07	0.05	0.14	0.19
Neighbourhood quality		0.81	0.33	0.61	0.53
Neighbourhood disorder			0.36	0.50	0.43
Social cohesion				0.31	0.25
Deprivation					0.54

(1) Higher scores on the different neighbourhood variables represent a more negative evaluation of the neighbourhood.

(2) Proportion of people reporting fair or poor health.

We found that a modest part of the variation in self-rated health can be found at the neighbourhood level (3.7%). Model 1 found strong associations between the different socio-demographic variables and self rated health (see Table 3). Respondents who were classified as skilled manual, partly and unskilled manual, and 'other' social class were more likely to report poor health. Similarly, people who were unemployed, retired, disabled, or with an 'other' employment status were more likely to report poor health. Non-homeowners were also more likely to report poor health. Model 2a shows the unadjusted associations between the neighbourhood variables and self rated health. Poor access to amenities, poor neighbourhood quality, neighbourhood disorder, a lack of social cohesion, and neighbourhood deprivation were all significantly associated with the reporting of poor health. Model 2b shows that the associations between the neighbourhood variables and self rated health were attenuated when controlling for socio-demographic differences. However, all associations remained significant. Model 2c additionally controlled for neighbourhood deprivation. All associations were further attenuated. The association between social cohesion and self rated health was rendered non-significant, as well as the association between neighbourhood disorder and self rated health.

**Table 3:** Unadjusted and adjusted odds ratios (OR) and 95% Confidence Intervals (CI) of reporting poor health

	*OR*	*95% CI*	*p*
**Model 1: Socio-demographics **^**(1)**^			
*Gender (female)*			
Male	1.10	0.99–1.21	0.075
*Age*	1.02	1.02–1.03	<0.001
*Social class (I & II)*			
III NM	1.07	0.93–1.24	0.352
III M	1.32	1.15–1.52	<0.001
IV & V	1.40	1.23–1.60	<0.001
Other	1.46	1.16–1.83	0.001
*Employment status (employed)*			
Unemployed	1.45	1.10–1.90	0.008
Retired	2.32	2.01–2.68	<0.001
Disabled	18.55	15.56–22.11	<0.001
Other	1.51	1.27–1.80	<0.001
*Household tenure (Homeowner)*			
Non-homeowner	1.57	1.39–1.76	<0.001
			
**Model 2a: Unadjusted **^**(2 3)**^			
Access to amenities	1.13	1.08–1.19	<0.001
Neighbourhood quality	1.33	1.27–1.39	<0.001
Neighbourhood disorder	1.25	1.19–1.32	<0.001
Social cohesion	1.14	1.08–1.20	<0.001
Deprivation	1.35	1.29–1.42	<0.001
			
**Model 2b: Adjusted for socio-demographics **^**(2 3)**^			
Access to amenities	1.10	1.04–1.15	<0.001
Neighbourhood quality	1.20	1.14–1.26	<0.001
Neighbourhood disorder	1.13	1.07–1.19	<0.001
Social cohesion	1.09	1.04–1.14	<0.001
Deprivation	1.19	1.13–1.25	<0.001
			
**Model 2c: Adjusted for socio-demographics and deprivation **^**(23)**^			
Access to amenities	1.07	1.02–1.12	0.004
Neighbourhood quality	1.13	1.07–1.20	<0.001
Neighbourhood disorder	1.05	1.00–1.11	0.059
Social cohesion	1.04	0.99–1.09	0.110

(1) Reference groups are given in brackets.

(2) The neighbourhood measures were included as z-scores, with higher values representing a more negative evaluation.

(3) The odds ratios are expressed for each SD increase.

Some interesting differences were found in the gender-stratified analyses (see Table 4). Model 1 shows that individual socio-economic status affected men and women differently. Unemployment significantly increased the odds of men reporting poor health, but did not do so for women. Model 2b shows that social cohesion was significantly associated with women's self-rated health, with a lack of social cohesion increasing the odds of women reporting poor health. However, social cohesion was not significantly associated with men's self-rated health. Remodelling the complete dataset with gender interactions terms showed that the interaction with unemployment was significant (OR = 1.82; 95%CI = 1.03–3.20); and that the interaction with social cohesion was marginally significant (OR = 1.07; 95%CI = 1.00–1.14). These interactions show that the odds of unemployed men reporting poor health are 82% higher than the odds of unemployed women reporting poor health; and that for each standard deviation change in neighbourhood social cohesion the odds of women reporting poor health are 7% higher as compared to the odds of men reporting poor health. No major gender differences were found for the contextual effects of access to amenities, neighbourhood quality and disorder, and neighbourhood deprivation.

**Table 4:** Odds ratios (OR) and 95% Confidence Intervals (CI) of reporting poor health for male and female respondents.

	*Male*			*Female*		
	*OR*	*95% CI*	*p*	*OR*	*95% CI*	*p*
**Model 1: Socio-demographics**^**(1)**^						
*Age*	1.02	1.02–1.03	<0.001	1.02	1.02–1.03	<0.001
*Social class (I & II)*						
III NM	1.03	0.79–1.34	0.834	1.05	0.88–1.25	0.610
III M	1.41	1.17–1.69	<0.001	1.19	0.93–1.52	0.159
IV & V	1.56	1.27–1.91	<0.001	1.29	1.08–1.54	0.005
Other	1.22	0.79–1.89	0.358	1.51	1.15–1.98	0.003
*Employment status (employed)*						
Unemployed	1.79	1.27–2.53	<0.001	1.00	0.62–1.62	0.984
Retired	2.44	1.97–3.03	<0.001	2.26	1.87–2.75	<0.001
Disabled	20.92	16.07–27.24	<0.001	17.59	13.83–22.37	<0.001
Other	1.53	1.04–2.25	0.032	1.48	1.22–1.81	<0.001
*Household tenure (homeowner)*						
Non-homeowner	1.57	1.30–1.89	<0.001	1.57	1.34–1.84	<0.001
						
**Model 2b: Adjusted for socio-demographics**^**(2 3)**^						
Access to amenities	1.10	1.03–1.18	0.007	1.09	1.03–1.16	0.003
Neighbourhood quality	1.20	1.12–1.29	<0.001	1.17	1.09–1.24	<0.001
Neighbourhood disorder	1.12	1.05–1.21	0.001	1.11	1.04–1.18	0.002
Social cohesion	1.05	0.97–1.13	0.221	1.12	1.06–1.19	<0.001
Deprivation	1.21	1.11–1.31	<0.001	1.16	1.08–1.24	<0.001

(1) Reference groups are given in brackets.

(2) The neighbourhood measures were included as z-scores, with higher values representing a more negative evaluation.

(3) The odds ratios are expressed for each SD increase.

## Discussion

This study examined the associations between different aspects of the neighbourhood environment and people's self rated health, and investigated whether these associations vary between men and women. The results showed that, before and after controlling for socio-demographic differences, the neighbourhood characteristics of poor access to amenities, poor neighbourhood quality, neighbourhood disorder, lack of social cohesion, and deprivation are associated with the reporting of poor health. They further showed that social cohesion is linked with women's health but not men's health, and that unemployment affects men's health but not women's health.

This study provides further empirical evidence for the idea that the neighbourhood environment is associated with people's health. It confirms the findings of a number of previous studies that the presence of neighbourhood problems and crime, and poor access to amenities, are linked with poor health [3-6,8,10]. The result that neighbourhood deprivation is detrimental to people's health, even when taking into account individual socio-economic status, is consistent with other studies on the impact of the socio-economic environment on public health [1,20]. Social cohesion was inversely related to the reporting of poor health, confirming that the social organisation of the local community plays an important role in public health [1,5,13,17,19,46]. Overall, the contextual effects of the different aspects of the physical and social environment could be considered modest, in particular when simultaneously controlling for socio-demographic differences and neighbourhood deprivation. In this context it has to be kept in mind that because of its large sample size the study has the statistical power to detect small neighbourhood effects. However, neighbourhood health effects are generally found to be small [5,6,17,28], and therefore large sample sizes are required to detect these effects. It also has to be considered that this study may have underestimated the size of neighbourhood effects through over-adjustment. While individual level variables are often seen as confounders, they could in some cases be conceptualized as mediators. That is, neighbourhoods may influence the life chances of individuals through their effects on achieved income and education, as well as through other residential segregation and selection processes [47].

The results of this study further suggest that the interpretation of the possible health effects of social and physical aspects of the environment may not be straightforward. We found that the health effects of neighbourhood quality, neighbourhood disorder, and social cohesion are substantially attenuated when controlling for individual and collective socio-economic status. This suggests that, instead of being aspects of the neighbourhood environment that independently contribute to people's health, they may mediate or reflect differences in socio-economic status. Unfortunately, the difference between mediation and confounding can not be shown using statistical methods alone, and a decision can only be made based on theory [48]. There is a need for more detailed analyses to determine the specific associations between neighbourhood deprivation and the quality of the social and physical environment, as well as the different possible pathways that link them to health. In either case, the results show that neighbourhood deprivation is associated with worse neighbourhood disorder, poorer neighbourhood quality, and less social cohesion.

The gender-stratified analyses show that a lack of social cohesion significantly increases the odds of women reporting poor health, but not the odds of men reporting poor health. In contrast, unemployment appeared more detrimental to men's than to women's health. These findings are similar to a number of recent studies, and suggest that whilst social aspects of the residential environment are more important for women, individual socio-economic status is more important for men [27-29]. It has been hypothesised that the neighbourhood environment is more important for women because they spend more time at home looking after children, doing domestic work or being primary carers [28]. Although we found that women were indeed more likely to look after the home and/or children, the contextual effects of neighbourhood social cohesion remained significant when adjusting for these differences. Others have suggested that time spent at home is an unlikely explanation, as there are no substantial gender differences in exposure to the residential environment [27]. Another possibility is that there are gender differences in the type and use of social relations [49]. It may be that men are more reliant upon workplace networks, and therefore are more affected by their employment status, but less so by the quality of the local environment. Indeed, men tend to report more support at the workplace than women, which seems to be caused by differences in occupational grade [50]. Although gender differences in the associations between neighbourhood and health have been demonstrated in a number of social epidemiological studies, there is a need to be cautious when interpreting these results. It remains unclear whether social interactions fulfil the same needs and/or mean the same thing to men and women.

It should be acknowledged that the current study has a number of limitations. These limitations are similar to most quantitative cross-sectional studies on neighbourhoods and health. Only associational claims can be made, as reverse causality can not be ruled out. Furthermore, the study focused on perceived aspects of the social and physical environment, which do not necessarily correspond with 'objective' aspects of the neighbourhood. In addition, and as a related issue, the study may have introduced a reporting or same source bias, as the area-specific measures were constructed using variables from the same study population as the health data. It would have been better to collect neighbourhood data independently from the main dataset [27]. However, that was not possible within the scope of this study. We have previously investigated whether same source bias was an important problem in the dataset [19]. In an analysis of associations between individual mental health status, small-area income deprivation and social cohesion, a split-half analysis of the dataset suggested that same source bias is not an important issue in this study.

In this study we used multilevel modelling to construct variables at the neighbourhood level of analysis. That is, we took the level 2 residuals of the neighbourhood perception variables to reflect the area-specific contributions to the overall scores. Although this alternative method of scale construction makes it difficult to compare the results with previous studies, the approach is preferable to the conventional method of aggregating individual level variables. The shrunken residuals derived from multilevel modelling provide more precise estimates of the different neighbourhood characteristics, as they only consider the part of the variance that is specifically attributable to neighbourhood differences.

Another methodological issue associated with many small-area studies is the use of administrative units as proxies for neighbourhoods. Identifying the correct geographical level of analysis is important, as misspecification may have implications for the study outcomes [47]. Although it is not clear whether EDs match people's perceptions of the neighbourhood, EDs are probably the best administrative units to represent neighbourhoods, as these are the smallest and most homogeneous geographical unit available. It may also be difficult to generalise the results to the larger (British) population. The study was conducted in one of the most deprived Welsh boroughs, and may not be representative of the wider population. However, it can also be considered a strength that the study was restricted to a well-defined geographical area. Considering that the residents of this borough share many characteristics and experiences it may be easier to draw conclusions on the basis of the identified neighbourhood effects. An additional strength of the study is the representative nature of the dataset obtained from a high response rate and high sampling fraction of individuals resident in Caerphilly borough. In this study the average response rate was 61.9% across the 325 EDs, and ranged from 32.7% to 87.5%. Although it is possible that the variation in response rate may have introduced a degree of response bias, we do not believe that it has substantially influenced the results of the study. This study's main aim was to examine associations between different area characteristics and health and not to make inferences about the study population itself, for which response rates are more important. Although it is likely that respondents with a lower socio-economic status were somewhat underrepresented in this study, this will only have affected the results if the effects of neighbourhood on health are different for this particular subgroup. Evidence for cross-level interactions between individual and neighbourhood socio-economic status is somewhat conflicting. Whereas some studies found an interaction between individual and area socio-economic status [51,52], other studies found no such cross-level interactions [53,54]. In addition, response rates were found to be weakly associated with neighbourhood deprivation, with response rates being slightly lower in neighbourhoods with a lower socio-economic status. This seems to suggest that we may have underestimated neighbourhood effects, as a lower response rate in deprived neighbourhoods may have reduced health variation across the different neighbourhoods.

The current study provides some pointers for health policy and practice. The findings that certain social and physical aspects of the neighbourhood environment are important for public health may help the design of community-based interventions. The results suggest that improvements in the environment, in terms of access to amenities, neighbourhood nuisances and neighbourhood disorder, may help to contribute to people's well being. However, this study also found that these aspects of the neighbourhood environment are strongly interlinked with socio-economic deprivation. As suggested before, this could mean that the aspects of neighbourhood quality and disorder, and social cohesion reflect differences in neighbourhood deprivation. Therefore the underlying socio-economic context should not be ignored when interpreting the results.

The results further contribute to the newly emerging evidence that there are important gender differences in the associations between the neighbourhood environment and health. Hitherto, only a small number of studies have examined gender differences in (contextual) neighbourhood health effects [26-29]. The results with regard to the social environment appear to be in line with those studies, despite differences in the conceptualisation and measurement of the social environment. For example, where Stafford et al. [27] used social trust measures that were collected in an independent sample, the current study used an ecometric approach to transform individual-level variables from the same dataset into contextual variables. Unlike Stafford et al., we found no significant interaction between gender and neighbourhood deprivation – even though we also obtained the deprivation indicator from an independent external source. Despite the absence of a statistically significant interaction between neighbourhood socio-economic status and gender, the results show that individual socio-economic status is more important for men than for women. Overall, the suggestion that men and women interact with their environment in a different way, and thus are likely to benefit from different types of interventions, may have important health policy implications. This could mean that health initiatives should be more community based for women and more workplace based for men. However, further research is needed to gain a more detailed understanding of the social processes underlying these gender health effects.

## Conclusion

Our results indicate that various aspects of the social and physical neighbourhood environment are associated with self rated health. However, these associations seem to be confounded by neighbourhood deprivation, suggesting that the neighbourhood aspects of access to amenities, neighbourhood quality, neighbourhood disorder, and social cohesion mediate or reflect differences in socio-economic status. The study further suggests that the social environment is more important for women's health, but that individual socio-economic status is more important for men's health. The suggestion that men and women benefit differently from the neighbourhood environment warrants further investigation of gender differences in neighbourhood health effects.

## Competing interests

The author(s) declare that they have no competing interests.

## Authors' contributions

DF designed the CHSNS and secured the funding. WP and DF conceived and carried out the analysis in this paper. DF and FD provided statistical advice. All authors read, critically revised, and approved the final manuscript.

## Pre-publication history

The pre-publication history for this paper can be accessed here:


